# Multicenter randomized controlled trial comparing digital and traditional chest drain in a VATS pulmonary lobectomy cohort: interim analysis

**DOI:** 10.1186/s13019-021-01567-y

**Published:** 2021-07-05

**Authors:** Paolo Mendogni, Davide Tosi, Giuseppe Marulli, Giovanni Maria Comacchio, Sara Pieropan, Veronica Rossi, Debora Brascia, Luigi Gaetano Andriolo, Giovanna Imbriglio, Gianluca Bonitta, Camillo Lopez, Federico Rea, Mario Nosotti

**Affiliations:** 1 Thoracic Surgery and Lung Transplant Unit, Foundation IRCCS Ca’ Granda Ospedale Maggiore Policlinico, Via Francesco Sforza, 35, 20122 Milan, Italy; 2 Thoracic Surgery Unit, Università degli Studi di Bari, Bari, Italy; 3 Thoracic Surgery Unit, Università di Padova, Padova, Italy; 4 Thoracic Surgery Unit, “Vito Fazzi” Hospital, Lecce, Italy; 5 Department of General and Specialistic Surgery “Paride Stefanini”, Policlinico Umberto I, University of Rome Sapienza, Rome, Italy

**Keywords:** Digital chest drainage, Water-seal chest drainage, Video-assisted thoracic surgery (VATS), Air leakage, Postoperative complications

## Abstract

**Background:**

The usefulness of digital chest drain is still debated. We are carrying out a study to determine if the use of a digital system compared with a traditional system reduces the duration of chest drainage. To evaluate safety, benefit, or futility of this trial we planned the current interim analysis.

**Methods:**

An interim analysis on preliminary data from ongoing investigator-initiated, multicenter, interventional, prospective randomized trial. Original protocol number: (NCT03536130). The interim main endpoint was overall complications; secondary endpoints were the concordance between the two primary endpoints of the RCT (chest tube duration and length of hospital stay). We planned the interim analysis when half of the patients have been randomised and completed the study. Data were described using mean and standard deviation or absolute frequencies and percentage. T-test for unpaired samples, Chi-square test, Poisson regression and absolute standardized mean difference (ASMD) were used. *P*-value < 0.05 was considered significant.

**Results:**

From April 2017 to November 2018, out of 317 patients enrolled by 3 centers, 231 fulfilled inclusion criteria and were randomized. Twenty-two of them dropped out after randomization. Finally, 209 patients were analyzed: among them 94 used the digital device and 115 the traditional one. The overall postoperative complications were 35 (16.8%) including prolonged air leak (1.9%). Mean chest tube duration was 3.6 days (SD = 1.8), with no differences between two groups (*p* = 0.203). The overall difference between hospital stay and chest tube duration was 1.4 days (SD = 1.4). Air leak at first postoperative day detected by digital and traditional devices predicted increasing in tube duration of 1.6 day (CI 95% 0.8–2.5, *p* < 0.001) and 2.0 days (CI 95% 1.0–3.1, *p* < 0.001), respectively.

**Conclusions:**

This interim analysis supported the authors’ will to continue with the enrollment and to analyze data once the estimated sample size will be reached.

**Trial registration:**

Trial registration number NCT03536130, Registered 24 May 2018 - Retrospectively registered.

**Supplementary Information:**

The online version contains supplementary material available at 10.1186/s13019-021-01567-y.

## Background

Air leaks are common after pulmonary resection, affecting patients with a prevalence ranging from 3 to 33%, and are associated with increased morbidity, prolonged hospital stay and increased costs [1, 2].

Conventionally, assessment of air leaks relied on the measurement of “bubbles in a chamber” by using traditional chest drainage systems. In recent years, novel digital devices have been introduced; it is believed that these devices have specific advantages. Digital drainages provide a continuous and objective assessment of air leaks, minimizing interobserver variability [3], thus reducing the need for tube clamping trials and finally optimizing the timing of chest tube removal. Moreover, these modern systems could be able to distinguish an active air leak from pleural space effect by evaluating the differential intrapleural pressure [4]. Finally, digital devices could help identify patients at high risk for prolonged air leak, allowing a better patient management in terms of either active intervention or early discharge from the hospital with a one-way valve system [5, 6].

Few studies, either retrospective or randomized controlled trials (RCT), comparing digital and analog chest drainage systems have been published, and results are conflicting regarding the advantages of one system over the other. In particular, it is not clear yet if the novel systems could actually lead to optimization of chest tube management in terms of chest tube duration and therefore length of hospital stay [7–9].

Considering that additional evidence is needed to further probe the potential clinical utility and impact of digital chest drainage devices, we implemented a randomized controlled trial; the title was “Comparison Between Electronic and Traditional Chest Drainage Systems” (NCT 03536130). This trial started in 2017; recruitment will finish by the end of 2020.

To evaluate safety, benefit, or futility of this RCT we planned the current interim analysis.

## Methods

This is the interim analysis on preliminary data from ongoing investigator-initiated, multicenter, interventional, prospective randomized trial. The interim main endpoint was overall complications within 30 days of the surgery; secondary endpoints were the concordance between the two primary endpoints of the RCT (chest tube duration and length of hospital stay).

**Fig. 1:**
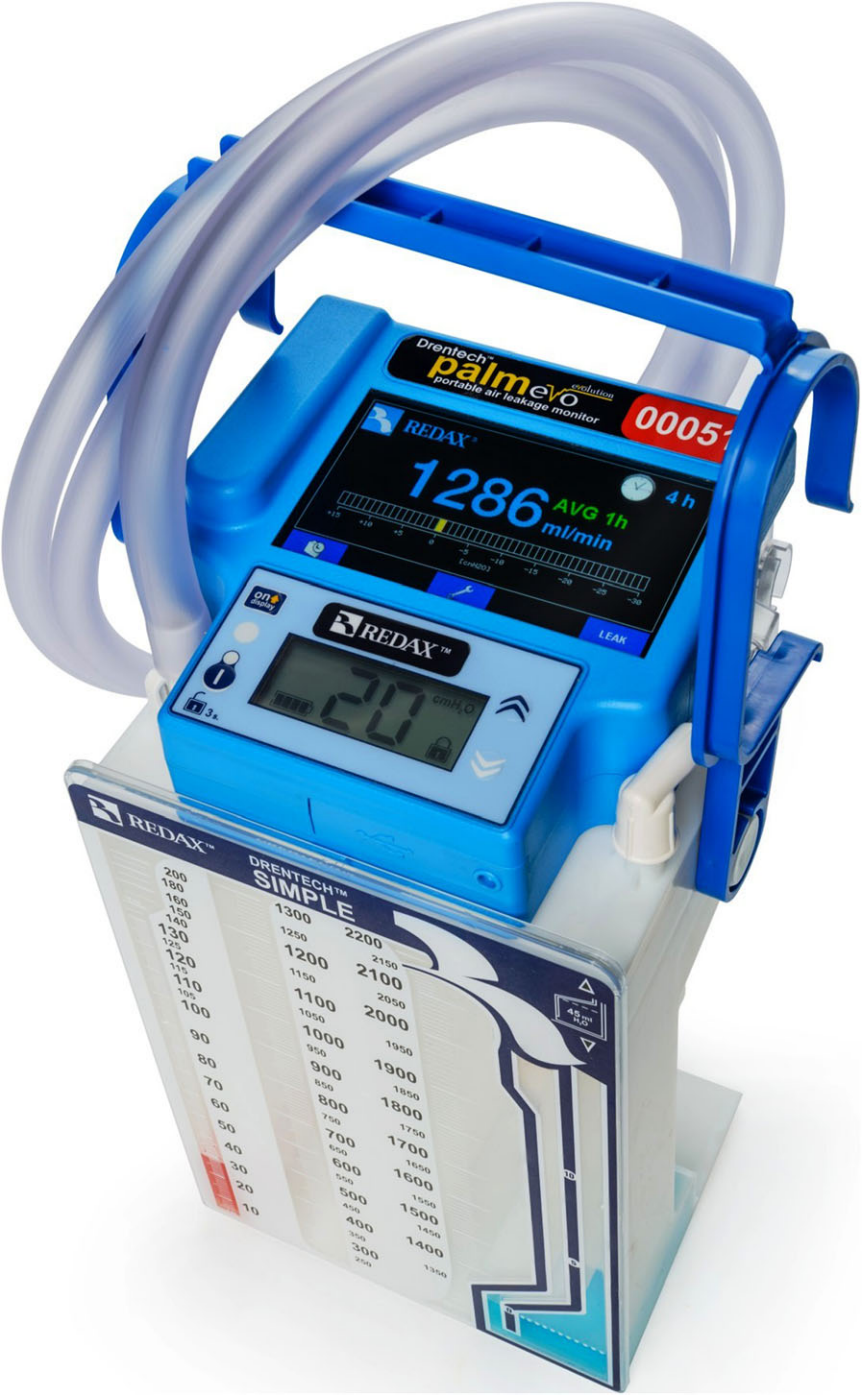
Chest drainage connected to digital device (Drentech™ Palm Evo system - Redax)

The RCT protocol was published online (clinicaltrials.gov/ct2/show/study/NCT03536130. Briefly, three different staff surgeons from three Italian high-volume thoracic surgery units enrolled all adult patients scheduled for video assisted thoracic surgery (VATS) lobectomy for both malignant and benign disease. All patients signed and dated an Italian-written informed consent form approved from ethical committees of the three hospitals. Individual randomization, stratified by centers, was performed with a 1:1 allocation to the intervention (Digital group) and control groups (Traditional group); the nature of the intervention did not allow blind randomization. At the end of surgery, a 28 Ch chest tube was connected to a digital device (Drentech™ Palm Evo system - Redax, Fig. 1) or traditional water-seal drain system. Chest tube was removed when chest X-rays show a complete lung expansion and there was no detectable air leak on traditional devices or when airflow is lower than 20 ml/min for at least 8 h on digital ones. In addition, daily fluid drainage should be less than 300 ml. When air leaks exceed 7 days they are considered “prolonged”. The sample size of the RCT is 382 patients (191 per group); the calculation was based on the two co-primary outcomes: duration of chest drain and length of hospital stay. The trial protocol was previously published meticulously detailing the study procedure [10]. We planned the interim analysis when half of the patients have been randomised and completed the study.

In this interim analysis, safety was measured by complication rates; complications were defined according the Common Terminology Criteria for Adverse Events v4.03 and they were recorded when classified as grade 2 or greater. The concordance between co-primary outcomes was measured by difference between hospital stay and chest tube duration (days). Benefit and futility were estimated by comparing the duration of drainage between the two groups (days).

The study adheres to CONSORT guidelines, and a completed CONSORT checklist is available as Supplementary Material.

### Statistical analysis

Data are presented as mean and standard deviation (SD). Categorical variables are shown as absolute frequencies and percentages. Student’s T and χ2 tests were performed as appropriate. The normality assumption was assessed by visual inspection of histogram and Q-Q plot. We used the absolute standardized mean difference (ASMD) to evaluate covariance balance between study groups; a value equal or less than 0.20 was considered as a small effect size. The Poisson regression was performed. The 95% Wald confidence intervals were computed. Two-sided *p*-value was considered statistically significant when < 0.05. All analyses were carried out using R version 3.2.2 software [11].

## Results

**Fig. 2:**
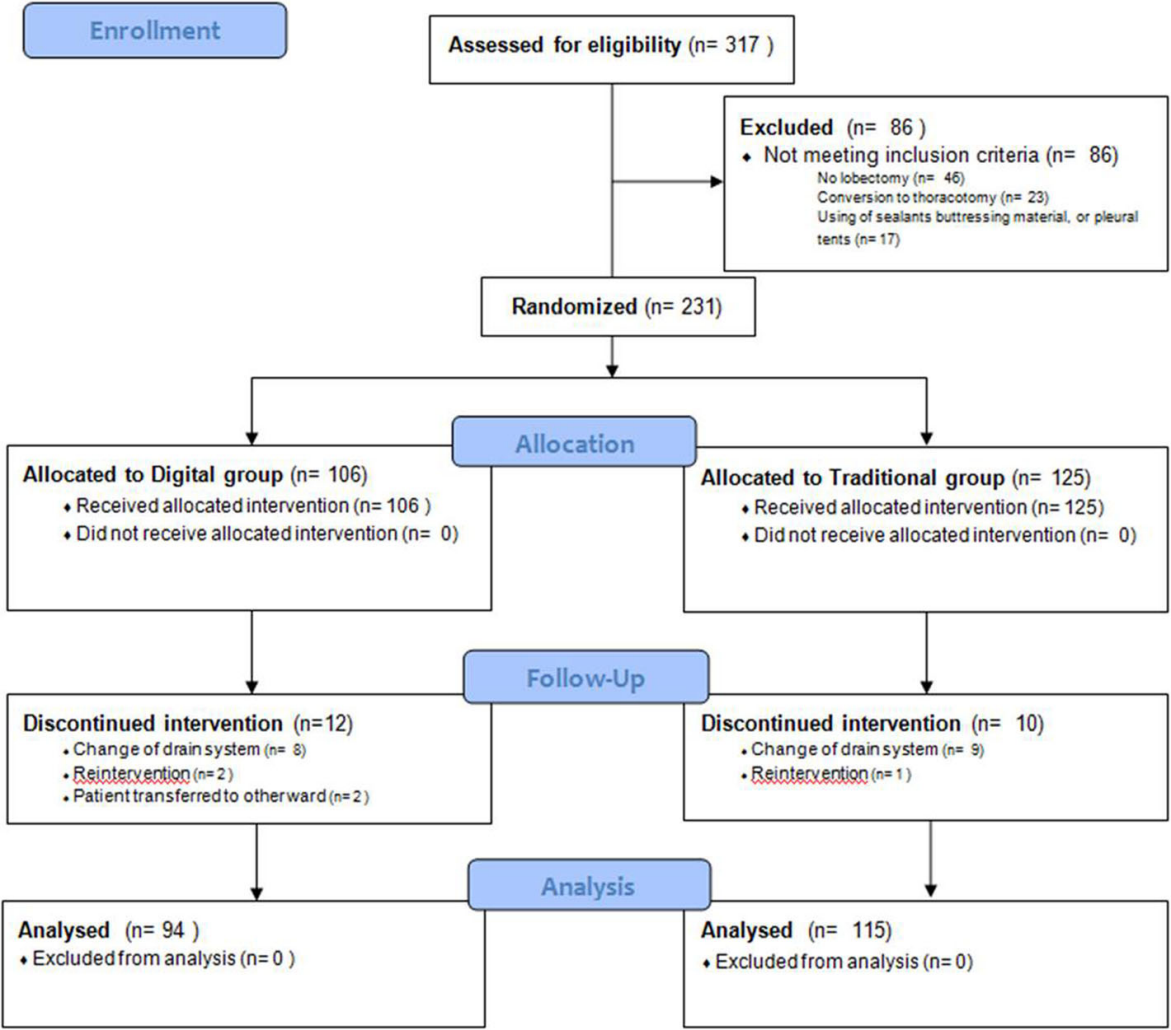
CONSORT Flow Diagram of the study

Three-hundred and seventeen patients who potentially met the inclusion criteria underwent lung resection between April 2017 and November 2018. CONSORT flow diagram summarizes patients’ recruitment (Fig. 2); finally, 209 patients were included in the current interim analysis: 94 assigned to Digital group and 115 to Traditional group. Table 1 shows preoperative and intraoperative patients characteristics; the comparison of the two groups with the ASMD did not overstep the value of 0.20 for any of the characteristics showing a substantial balance of the covariates. The overall postoperative complications were 35 (16.8%) including prolonged air leak (1.9%); Table 2 shows the results distributed per group; none of the described complications could be correlated with the drainage devices. Mean chest tube duration was 3.6 days (SD = 1.8); there were no statistically significant differences between groups: difference of means was − 0.33 days (95% CI: − 0.83 to − 0.018; *p* = 0.203). The overall difference between hospital stay and chest tube duration was 1.4 days (SD = 1.4).

**Table 1:** Preoperative and intraoperative patients’ characteristics

	Digital group (***n*** = 94)	Traditional group (***n*** = 115)	ASMD
**Age, years, mean (SD)**	67 (9)	66 (11)	0.10
**Male, n (%)**	57 (60.6)	61 (53.0)	0.15
**Co-morbidity, n (%)**	68 (72.3)	89 (77.4)	0.05
**COPD, n (%)**	15 (16.0)	12 (10.4)	0.17
**Asthma, n (%)**	3 (3.2)	3 (2.6)	0.04
**FEV1, %, mean (SD)**	96.6 (20.2)	98.9 (20.4)	0.11
**FVC, %, mean (SD)**	101.6 (19.9)	103.9 (18.9)	0.12
**Tiffeneau, %, mean (SD)**	77.7 (9.7)	76.8 (12.0)	0.08
**DLCO, %, mean (SD)**	83.1 (18.4)	81.6 (19.0)	0.08
**Induction CT, n(%)**	6 (6.4)	3 (2.6)	0.18
**Induction RT, n(%)**	0 (0.0)	0 (0.0)	0.0
**Surgical time, min, mean (SD)**	164 (67)	169 (65)	0.08
**Hemostatic devices, n (%)**	49 (52.1)	52 (45.2)	0.14
**Type of lobectomy, n (%)**			
Right upper lobectomy	24 (25.5)	35 (30.4)	0.11
Right middle lobectomy	6 (6.4)	8 (6.9)	0.00
Right lower lobectomy	15 (15.9)	15 (13.0)	0.08
Left upper lobectomy	32 (34.0)	28 (24.3)	0.20
Left lower lobectomy	17 (18.1)	29 (25.2)	0.15
**Lymphadenectomy, n (%)**	93 (98.9)	113 (98.3)	0.05
Systematic	72 (76.6)	86 (74.8)	0.04
Sampling	21 (22.3)	27 (23.5)	0.03

*ASMD* Absolute standardized mean difference, *SD* Standard deviation, *COPD* Chronic obstructive pulmonary disease, *FEV1* Forced expiratory volume at 1 s, *FVC* Forced vital capacity, *DLCO* Diffusion Lung CO, *CT* Chemotherapy, *RT* Radiation therapy. No significant differences were observed (*p* > 0.05)

**Table 2:** Postoperative events

	Digital group (***n*** = 94)	Traditional group (***n*** = 115)	***p*** value
**Total complications, n (%)**	17 (18.1)	18 (15.6)	0.999
Cardio-vascular complications, n (%)	6 (6.4)	6 (5.2)	0.951
Pulmonary complications, n (%)	7 (7.4)	11 (9.6)	0.767
Other, n (%)	4 (4.3)	2 (1.7)	0.505
**Prolonged air leak, n (%)**	1 (1.1)	3 (2.6)	0.999
**Chest tube duration, days, mean (SD)**	3.4 (1.8)	3.8 (1.8)	0.999
**Difference between hospital stay and chest tube duration, days (SD)**	1.3 (1.0)	1.4 (1.7)	0.999

The mean duration of chest tube drainage in patients with POD1 air leaks was 4.4 days (SD = 1.8 days) and 5.1 days (SD = 1.9 Days) in Digital group and Traditional group respectively (*p* = 0.175).

The mean duration of chest tube drainage in patients without POD1 air leaks was 2.8 days (SD = 1.3 days) and 3.3 days (SD = 1.4 days) in Digital and Traditional groups, respectively (*p* = 0.022).

Air leak at first postoperative day detected by digital and traditional device predicted increasing in tube duration of 1.6 day (CI 95% 0.8–2.5, *p* < 0.001) and 2.0 days (CI 95% 1.0–3.1, *p* < 0.001), respectively.

## Discussion

The RCT “Comparison Between Electronic and Traditional Chest Drainage Systems” aims to identify the possible benefit of electronic drainage for managing patients who underwent VATS lobectomy. We regarded Data and Safety Monitoring Committee as unnecessary, considering that the device is commercially available and the device was approved by the Ministry of Health; nevertheless, we planned the interim analysis to assess safety, benefit or futility.

This RCT had a dropout rate of 9.5%, which was close to the median percentage of patients with a missing outcome from RCTs published in 4 top medical journals recently reviewed [12]. We considered the missing data as “missing completely at random”; we are aware that this is a strong assumption but, on the other hand, the intention-to-treat principle was impossible to apply due to the very nature of this RCT. We have not recorded any unfavorable events directly attributable to the drainage devices, whether they are digital or not. Postoperative complications were similar between the two groups and not directly related to the type of device. We can affirm that the study protocol did not cause any harm to the enrolled patients and therefore proved to be safe.

The difference between the two groups in terms of chest tube duration was not statistically significant, thus the study cannot be stopped for benefit. However, a trend toward shorter chest tube duration and length of stay was observed in the Digital group; as a consequence, we consider it cost-effective and justified to continue the study to determine if this trend will reach statistical significance.

Finally, two additional items worth some comments: the primary objective and a predictive factor for prolonged air leak. Our RCT has a primary composite objective, therefore we wanted to check that the two parameters (duration of chest tube drainage and length of hospital stay) were actually correlated; we assumed that the difference between the two parameters should remain within 2 days. Indeed, the data collected in this interim analysis showed that the two parameters remained closely related and consequently a re-modulation of the study protocol will not be necessary. Despite this study was not planned to identify prognostic factors for air leak, we observed that the presence of air leakage on the first postoperative day predicted the prolonged chest tube requirement as reported by others [13].

## Conclusions

In conclusion, this interim analysis supported the authors’ will to continue with the enrollment and to analyze data once the estimated sample size will be reached.

## Supplementary Information

**Additional file 1.**

## Data Availability

The datasets generated and analyzed during the current study are available from the corresponding author on reasonable request.

## Abbreviations

ASMD: Absolute standardized mean difference
CI: Confidence interval
RCT: Randomized controlled trial
SD: Standard deviation
VATS: Video assisted thoracic surgery
